# Effects of Electric Field Dimensions on Electrokinetically Enhanced Cadmium Dissociation and Phytoremediation in Plateau Red Soil

**DOI:** 10.3390/plants15030481

**Published:** 2026-02-04

**Authors:** Lirong Wang, Ming Zhao, Zhengyang Duan, Feipeng Qin, Hongyan Ma, Yuchao Zhu, Ming Jiang, Xuan Zhu, Tianguo Li

**Affiliations:** 1 College of Resources and Environment, Yunnan Agricultural University, Kunming 650201, China; wanglirong6663@163.com (L.W.); 18637852141@163.com (M.Z.); 15398525349@163.com (F.Q.); 2020210273@stu.ynau.edu.cn (H.M.); 18168339488@163.com (Y.Z.); mingjiang2010@163.com (M.J.); 2 College of Resources, Environment and Chemistry, Chuxiong Normal University, Chuxiong 675000, China; zyduan@cxtc.edu.cn

**Keywords:** electrokinetic–assisted phytoremediation, electric field dimensions, periodic reversal, soil section, Cd accumulation

## Abstract

The enhanced performance of electrokinetics (EK) on the cadmium (Cd) dissociation, redistribution, and phytoremediation of Cd-contaminated agricultural soil has been investigated based on the application of an electric field in different dimensions (1D, 2D, 3D). In electrokinetic–assisted phytoremediation (EKPR), unlike the uniform pH change observed in 1D treatment, more soil points (P1–P9) under 2D/3D electric fields were exposed to the influence of the anode (or cathode during polarity switching). *Sedum plumbizincicola* mitigates EK-induced soil acidification and alkalization, particularly anode acidification under high voltage (10–20 V). Studies reveal that EK promotes Cd dissolution into soil pore water, with a 227.82% maximum increase in the anode region under EK2 treatment of 10 V voltage, facilitating Cd phytoextraction. Periodically reversed DC electric fields enhanced *Sedum plumbizincicola* height more significantly than biomass, with no conspicuous regional differences. Overall, EKPR (voltage of 5–10 V) can effectively promote soil Cd phytoremediation due to the synergistic effect of direct interface action and indirect influence of the electric field to improve the Cd speciation evolution, dissociation, and bioavailability at the soil–water interface. The appropriate electric field arrangement and voltage were 2D treatment (EKPR2) and 5 V for *S. plumbizincicola*, respectively. In this case, the average Cd removal rate was as high as 50.23%, and the biomass and Cd accumulation increased by 16.59% and 29.31%. This suggests that plant growth constitutes the pivotal stage driving Cd accumulation and ultimately achieving Cd removal from soil, which is the key to enhancing remediation efficiency. Meanwhile, the configuration and intensity regulation of electric fields, as core elements ensuring the enhanced efficacy of electrokinetic–assisted phytoremediation (EKPR), can indirectly affect plant growth and Cd accumulation processes by modulating intermediate variables such as soil pH, nutrient status, and heavy metal speciation evolution.

## 1. Introduction

Soil heavy metal pollution has become a global environmental problem [1]. As far as China is concerned, about 2.786 × 10^9^ m^2^ of agricultural soil is contaminated by Cd [2,3]. The remediation of Cd-contaminated soil is a matter of priority to safeguard agricultural production and food safety. Common contaminated soil remediation techniques include physical, chemical, and biological methods [1,4]. Among them, physical techniques such as replacement, vitrification, and solidification have large engineering volumes, high cost, and energy consumption, while chemical techniques such as oxidation, reduction, and adsorption easily produce secondary pollutants and cause large soil disturbance [5,6,7]. Therefore, cost-effective and environmentally friendly bioremediation has been widely studied [8].

Phytoremediation is a highly efficient and in situ-applicable soil bioremediation technology, which uses the accumulation behavior of heavy metals by plants to absorb or stabilize heavy metals in soil [9]. *Sedum plumbizincicola*, as a Cd hyperaccumulator, has been widely used in the remediation of Cd–contaminated soil due to its rapid growth rate and large biomass [10,11]. However, the roots of *S. plumbizincicola* are shallow, and it can only uptake Cd with high availability within the reach of roots; the remediation efficiency is limited by the accessibility and bioavailability of Cd in the actual contaminated soil [12]. Electrokinetic–assisted phytoremediation (EKPR) involves the application of low-intensity electric fields to stimulate plant growth or promote the absorption of heavy metals by promoting the desorption and short-distance transport of heavy metals [10,13]. During the initial research phase, EKPR was only feasible for demonstration in sites with high heavy metal concentrations and high ecological risks. This limitation was primarily attributed to the complex design of electrode arrays and substantial energy consumption of conventional power supply modes, which resulted in prohibitive costs for large-scale applications and confined the technology to high-priority remediation scenarios. In recent years, the large-scale adoption of renewable energy sources and the integration of photovoltaic agriculture complementary technologies have significantly lowered energy costs and mitigated engineering implementation difficulties, thereby overcoming the inherent technical and economic barriers. This progress has expanded the applicability of EKPR from high-concentration contaminated sites to low-to-moderate concentration agricultural soils, which are more relevant to agricultural production and food security. In a 3-year pilot study conducted on Cd-contaminated paddy soil in the southern Chinese province of Guangdong, the deployment of 0.8–1.2 m vertical electrodes resulted in a 42–58% reduction in bioavailable Cd without compromising rice cultivation [14]. Furthermore, 3D electrokinetic phytoremediation systems have demonstrated efficacy in the remediation of Pb/Cd-contaminated industrial brownfield sites, meeting the criteria for land reuse [15]. These practical cases have accumulated valuable experience for the practical engineering application of electrokinetic remediation technology and provided a certain reference for the further exploration and practice of this technology in the remediation of similar contaminated sites. Current EKPR research focuses on assessing the feasibility of technologies, optimizing parameters, and overcoming adverse effects [16,17,18,19]. The selection and application of electric field conditions (electric field type and arrangement, electric field intensity, and application time), soil pH control (periodic reversal, alternating electric field, and buffer pair) and additives (electrolyte, plant nutrients, soil conditioner, pH regulator, and heavy metal chelating agent) have significant effects on the desorption and migration ability, spatial distribution, and plant accumulation characteristics of soil heavy metals [10,20].

Previous studies have shown that the suitable electric field intensity for EKPR is 0.5–4.0 V cm^−1^. However, the application of a direct current (DC) electric field (more than 4.0 V cm^−1^) for a long time will lead to excessive acidification and alkalization of soil, reduce the number of microorganisms, and cause damage to plants [21,22]. In order to alleviate the above adverse effects, the application of periodic reversal of DC or AC (alternating current) electric field and pH regulation (adding organic acids, introducing electrolytes with pH buffer regulation function, or electrolyte circulation) is currently the main mitigation measure [23]. However, it should be noted that the introduction of electrolyte solutions causes impregnation of soil pores and particles, thereby inducing soil acidification or alkalization effects [24], which is not conducive to the growth of most plants. Therefore, the mode of directly embedding electrodes in soil may be more suitable for plant growth and the practical application of EKPR technology. Periodic reversal serves as an effective strategy to prevent soil pH polarization. By cyclically exchanging electrodes, soil pH can be dynamically regulated to mitigate extreme acidic or alkaline conditions. Concurrently, electroosmosis, electromigration, and electrochemical reactions promote the activation of heavy metals, which optimizes the electrokinetic remediation environment [25]. Among these processes, the impacts of electrokinetic effects on the transformation of heavy metal forms and their distribution at the soil–water interface are directly related to the efficiency of the EKPR system in treating actual contaminated soils, which needs further study. Although this electric field configuration enhances plant biomass and metal uptake efficiency, systematic investigations into the desorption, migration, distribution, and transformation mechanisms of heavy metals under its influence remain scarce [19,22]. This constitutes one of the bottlenecks constraining the large-scale application of EKPR.

To our knowledge, EKPR is involved in the complex interactions of chemical and electrochemical processes at the soil–water interface [4,26]. Whether other mechanisms are included is worth exploring to elucidate the mechanism and regulation of the EKPR system. In this study, the actual Cd-contaminated agricultural soil, *S. plumizincicola*, and Cd were taken as research objects. The DC electric field with direct connection and periodic reversal of electrodes was constructed, and the EKPR pot experiment with three electric field dimensions (1 D, 2 D, and 3 D) and voltage intensity (5 V, 10 V, and 20 V) was set. The main objectives of this study were (1) to describe soil pH changes; (2) to analyze the spatial distribution, chemical speciation changes, and desorption behavior of Cd; (3) to track the growth and Cd accumulation of *S. plumbizincicola*; (4) to explore the relationship between Cd desorption and the influence factors of soil pH and electric field. The mechanism and regulation of the EKPR system were clarified, the reasons for the promotion of Cd accumulation in plants under electrical action were revealed, and the effects of various combinations of electric field configurations were evaluated.

This study aims to clarify the mechanism and regulatory rules of the EKPR system, and to reveal the intrinsic relationship between soil pH value, Cd desorption behavior, plant growth, and Cd accumulation under the influence of an electric field. This can enrich the theoretical system of the interaction between the electric field and the soil–plant–metal system, fill the research gap of the multidimensional electric field regulation mechanism in the EKPR remediation process, and provide important theoretical support for the in-depth development and innovation of electric field-assisted plant remediation technology.

## 2. Methods

### 2.1. Materials

The *S. plumbizincicola* seedlings were obtained from the farmland soil restoration base of Lanping Jinding lead-zinc mining area, Nanjing Institute of Soil Research, Chinese Academy of Sciences. Following one month of cutting propagation, the well-grown seedlings with a height of approximately 10 cm were selected for the pot experiment. The contaminated agricultural soil that was the subject of the study was obtained from Zhehai Town, Huize County, Qujing City, Yunnan Province, China (N 26°34′7.46″, E 103°38′19.72″; with an average altitude of 2124 m), a representative heavy metal-polluted region in Yunnan Province. Following air-drying, the soil samples were subjected to a 2 mm aperture sieve for utilization. Table S1 shows soil physical and chemical properties.

### 2.2. Pot Experiment Design

**Figure 1:**
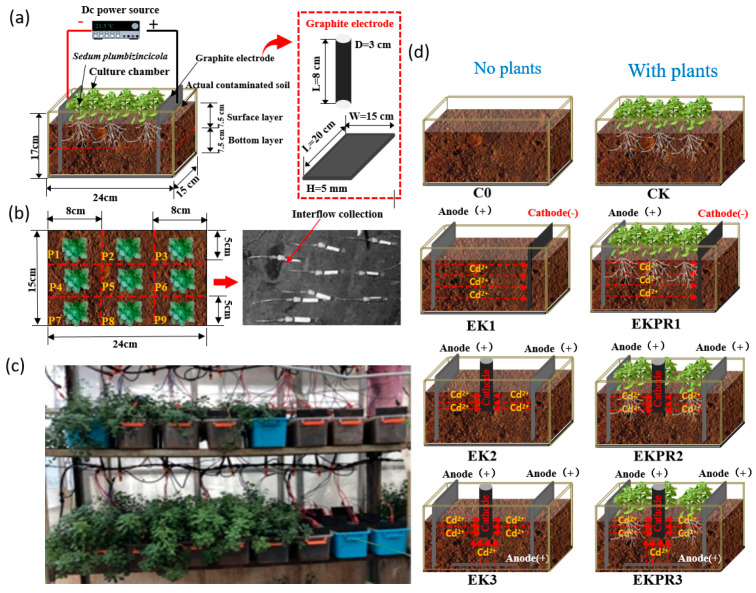
A diagram of the EKPR system: (**a**) a diagram of the EKPR device and graphite electrode; (**b**) P1–P9 points and soil pore water collection diagram; (**c**) EKPR pot experiment site situation; (**d**) control and three kinds of electric field layout diagrams.

The pot experiment was conducted in the greenhouse of the eastern campus of Yunnan Agricultural University. A rectangular PVC culture basin (length × width × height = 2.4 × 1.5 × 1.7 dm) (Figure 1a) was used for each experiment, and each basin was filled with 5 dm^3^ of contaminated soil. The electrode materials used in this study were a graphite electrode plate (length × width × height = 2.0 × 1.5 × 0.05 dm) and a graphite electrode rod (length: 0.8 dm, diameter: 0.3 dm) (Figure 1a). The electrode system was powered by a DC power supply (0–100 V, 0–3 A). To accurately investigate the speciation and spatial distribution characteristics of Cd at different soil positions, the pot was divided into 9 sampling regions of equal area by establishing a 3 × 3 grid with the center of the basin as the origin. These regions were labeled P1 (top-left), P2 (top-middle), P3 (top-right), P4 (middle-left), P5 (central), P6 (middle-right), P7 (bottom-left), P8 (bottom-middle), and P9 (bottom-right), and one *S. plumbizincicola* was planted in each point (Figure 1b,c). This experiment established six remediation systems (Figure 1d): one-dimensional (1D) electric field electrokinetic remediation system (EK1), 1D electric field electrokinetic-plant co-remediation system (EKPR1), two-dimensional (2D) electric field electrokinetic remediation system (EK2), 2D electric field electrokinetic-plant co-remediation system (EKPR2), three-dimensional (3D) electric field electrokinetic remediation system (EK3), and 3D electric field electrokinetic-plant co-remediation system (EKPR3). Each system employed three voltage settings (5 V, 10 V, 20 V), and the phytoremediation control system (CK) without an electric field was constructed. Each treatment was replicated three times, with a total of 57 basins (Table S2). Different dimensional electric fields were achieved via differentiated electrode arrangements: A one-dimensional (1D) electric field was generated by placing a graphite electrode plate at each end along the longitudinal direction of the culture basin (serving as the anode and cathode, respectively), thus forming a linear electric field extending along the basin’s longitudinal axis. A two-dimensional (2D) electric field was constructed by positioning a graphite electrode plate (acting as an anode) at each longitudinal end of the culture basin, with a graphite electrode rod (acting as a cathode) placed at the basin center, thereby establishing a two-dimensional planar electric field. A three-dimensional (3D) electric field was established by placing a graphite electrode plate (all acting as anodes) at both longitudinal ends and the bottom of the culture basin, with a graphite electrode rod (acting as the cathode) positioned at the basin center, thus forming a three-dimensional spatial electric field. In addition, a control group C0 (no plants, no electric field application) was set up. Neither C0 nor CK (the non-electric field control group) was subjected to grid partitioning. Approximately 500 mL of water was poured into each basin every 3 days. Electricity was applied daily for 8 h (9:00–17:00), with electrode polarity reversed every 7 days. The treatment was conducted in cycles of 14 days, and a total of 10.5 cycles were applied, corresponding to an overall experimental duration of approximately 150 days. For the EKPR process, three soil pore water sampling points were set at the mid-position of the four equidistant points in the pot (i.e., 5 cm, 10 cm, and 15 cm from the anode). During the vigorous growth stage of *S. plumbizincicola*, a total of three pore water samples were collected over three consecutive energization half-cycles (21 d), with one sample taken every seven days after each polarity reversal. For the same batch, the pore water samples from the three positions were amalgamated to form a composite pore water sample for each treatment. The pore water of soil was collected in situ by means of a special RHIZONMOM pore water sampler (Rhizosphere Research Products BV, Wageningen, The Netherlands) of the Dutch type (Figure 1b). The sampling head was composed of a white porous hydrophilic filter membrane (length 10 cm, diameter 2.5 mm, average pore size 0.15 um, with reinforcement wire). A negative Luer connector (Wuxi NEST, Wuxi, China) was connected to a syringe for negative pressure collection. In the final week of the experiment, soil and *S. plumbizincicola* were collected according to the predefined grid sampling points (potted plant sampling was performed at the mid-phase of the final experimental cycle under polarized conditions, and the relative electrode zoning of Pots P1 to P9 was classified in accordance with the cathode and anode of the electric field applied in the final stage).

### 2.3. Research Method

Following the EK and EKPR experiments, *S. plumbizincicola* plants were collected from different regions. The plant height was measured, and then, they were harvested and separated into shoots and roots. The fresh and dry weights of both the shoot and root fractions were measured. After drying and grinding, the Cd content in the plant samples was determined by an atomic absorbance spectrometer (AAS–ICE3300, Thermo Fisher Scientific, Munich, Germany) after HClO_4_−HNO_3_ digestion [10]. Soil samples were collected from all points (P1–P9), naturally dried, and then ground through a 20-mesh sieve. Soil pH was determined via the water extraction (soil–water ratio 1:2.5 (*w*/*v*)) [27], pH meter (pH–3b, Shanghai Rex Instrument Factory, model, Shanghai, China). The total Cd of soil was determined after digestion by three acids (HNO_3_-HF-HClO_4_) and dilution with 1.0% HNO_3_ solution. The Cd content of pore water was determined after digestion by HNO_3_-H_2_O_2_. The modified BCR continuous extraction technique was used to analyze the soil Cd speciation [28]. Finally, the Cd content was determined by AAS. All chemicals utilized in the experimental procedure were of analytical grade.

### 2.4. Data Analysis

Excel was used for statistics and the calculation of each measurement index. SPSS 25.0 was used for significant difference analysis (*p* < 0.05) and correlation analysis. Origin 8.0 and Adobe Illustrator 2023 were used for drawing.

Pearson correlation analysis was employed to quantitatively assess the linear correlation among variables, with results presented as a heatmap. Mantel analysis was used to further verify the significance of the overall relationships between variable groups and identify the key soil factors influencing plant growth and Cd uptake under electrokinetic assistance. A partial least squares path model (PLS-PM) was constructed using the “plspm” package (version ≥ 0.4.9) in R software (version ≥ 4.0.0) to dissect the direct and indirect effects of electrokinetic field configuration and voltage intensity on plant Cd accumulation by altering soil physicochemical properties and Cd fraction distribution.

The relative migration rate (RMr) was used to characterize the migration of Cd in space. The accumulation characteristics of Cd in plants were expressed by bioconcentration factors (BCF) and translocation factors (TF). The removal efficiency of Cd in soil was expressed by η (whole basin), and the calculation formulas were as follows:

(1) $$RMr=\frac{C_{cz}\left({or\;C}_{Iz}\right)-C_{az}}{2\times C_{0}}\times 100\%$$

(2) $$BCF=\frac{C_{\;aboveground}}{C_{soil}}$$

(3) $$TF=\frac{C_{shoot}}{C_{root}}$$

(4) $$\eta =\frac{C_{0}-C_{e}}{C_{0}}\times 100\%$$

In the formulas, (1) C_cz_, C*_Iz_*_,_ and C*_az_* represent the average Cd content (mg⋅kg^−1^) in the soil of the cathode region, middle region, and anode region, respectively. C_0_ is the original Cd content (mg⋅kg^−1^) in heavy metal contaminated soil; (2) C*_aboveground_*, C*_shoot_* and C*_root_* represent the average Cd accumulation (mg⋅kg^−1^); (3) C*_soil_* represents the average Cd content (mg⋅kg^−1^); (4) C*_e_* is the soil Cd content after the experiment (mg⋅kg^−1^).

## 3. Results and Discussion

### 3.1. The Changes of Soil pH

**Figure 2:**
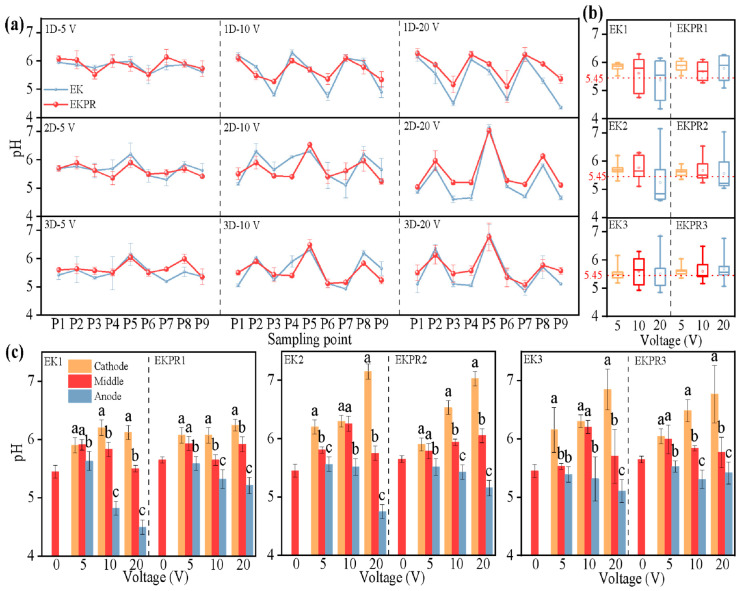
Soil pH under different electric fields: (**a**) pH of P1–P9 points; (**b**) a box plot of soil pH; (**c**) overall variation ranges of soil pH described in cathode, middle, and anode regions after integrating the region of P1–P9 applying the same rules. (Bars represent means ± S.E. of three independent replicates. Those not sharing the same letters are significantly different at *p* < 0.05, as determined by Duncan’s multiple range tests.)

Soil pH affects heavy metal remediation efficiency by influencing heavy metal speciation, spatial distribution, and plant growth. In this study, an attempt was made to systematically investigate the relationship between soil pH changes, electric field arrangement, and voltage from the perspective of different points (P1–P9) in the lateral (Figure 2a–c) and vertical (Table S3, voltage of 5 V) profiles of soil in the pots. As shown in Figure 2a, both the value and distribution of soil pH in different soil points varied significantly after long-term application of a periodic reversal DC electric field (150 d for 10.5 cycles). The degree of soil acidification and alkalization change at points P1–P9 is positively correlated with voltage, and indicated voltage is the dominant driving factor of soil pH change in EK and EKPR systems [25]. The soil pH at points P1–P9 after EK and EKPR treatment presented different distribution and variation rules due to the influence of different electric field dimensions (1D, 2D, 3D). But under the same electric field arrangement, the pH changes were consistent for the same region of P1–P9 with the increase in voltage. For 1D treatments (EK1, EKPR1), the soil pH showed a uniform distribution in the cathode region (P1, P4, P7), the middle region (P2, P5, P8), and the anode region (P3, P6, P9). But the soil pH presented a different distribution in the cathode region (P5), middle region (P2, P8), and anode region (P1, P3, P4, P6, P7, P9) for 2D and 3D treatments. In this case, in general, neither EK nor EKPR treatment made the soil pH extremely acidic and alkaline (4.35–7.15 vs. 5.45) due to the soil buffering capacity and periodic change (electrode reversal time was 7 days) of the DC electric field direction.

In order to clearly analyze the overall variation ranges of soil pH under different electric field intensities, Figure 2c integrates and describes the variation of soil pH under different treatments according to the above pH zoning rules. As expected, consistent with other literature reports, the pH decreased in the anode regions and increased in the cathode regions with the increase in voltage, but the middle regions showed no regular change trends in different electric fields. From Figure 2b,c, the soil pH change degree of EKPR treatment was effectively reduced, especially the degree of anodic acidification, compared to that of EK treatment. In terms of electric field arrangements, the cathode alkalization effect of the 2D and 3D electric fields was more obvious than that of 1D, whereas the anodic acidification effect was just the opposite. It is not difficult to see from the median, average, and quartile values of the box plot for EK treatment (Figure 2b) that although the largest soil pH appears (7.03–7.15 of 20 V) in the cathode region of 2D and 3D electric fields due to to more denser electric field lines [25,29], the overall acidification effect is even more pronounced. This is mainly attributed to the larger anode area and voltage strength [10,30]. Another contributing factor is that the migration rate of H^+^ in soil is higher than OH^–^. Under the electrodynamic effect, the OH^−^ generated at the cathode reacts with free divalent metal cations (e.g., Cd^2+^, Ca^2+^, Mg^2+^) in the soil solution to form insoluble hydroxides (e.g., Cd(OH)_2_, Ca(OH)_2_), resulting in the consumption of OH^−^ [31]. The synergistic effect of the electric field and planting *S. plumbizincicola* in EKPR contributed to the effective alleviation of this acidification phenomenon. EKPR resulted in a slight increase in overall soil pH under various electric field conditions, especially for low voltage (≤10 V). In this acid red soil (original pH is 5.45), the mean soil pH of CK slightly increased (5.65 vs. 5.45), and there was no obvious change in soil pH in each region. This phenomenon stands in opposition to the findings of Fan [10], who reported that plant *S. plumbizincicola* exhibited a marginal decline in the mean pH of surface soil (7.03 vs. 7.54). These results indicated that *S. plumbizincicola* can alleviate soil pH deterioration and mitigate acid–base imbalances by modulating root exudate secretion [32].

For a multidimensional electric field, this study investigated the pH change in the vertical direction and under the voltage of 5 V. There is no difference in pH in the surface and bottom soil under the 1D electric field, as shown in Table S3, while there is a significant difference in soil pH in the vertical direction of the 2D and 3D electric fields, which followed the order of surface layer > bottom layer. It is in line with the previous study results that in the 2D electric field, the soil pH of the bottom layer < middle layer < surface layer [10]. The plant root is generally distributed in the surface layer of the soil, where a too low pH will affect plant growth, but a lower pH is more favorable for heavy metal desorption [17]. The soil pH of the surface layer > bottom layer under a multidimensional electric field made it possible for plants to accumulate more heavy metals by promoting plant growth rather than inhibiting it. Heavy metals will be desorbed in the bottom layer with a lower pH, which will be migrated to the surface layer by controlling the direction of the electric field to be accumulated by the plant [33]. In addition, the present study found that the maximum pH difference between the surface and bottom soil layers was observed under the 2D electric field condition, contrary to the expectation that a larger vertical pH difference would be observed with increasing electric field dimensionality. This result may be related to the fact that the denser the electric field lines, the more pronounced migration of OH^–^ and H^+^ [18].

### 3.2. Soil Cd Content, Speciation and Spatial Redistribution Characteristics

The dissociation, transformation of chemical speciation, and spatial migration of heavy metals in soil significantly affect the contact probability between plant roots and metal ions, as well as the phytoextraction efficiency of phytoremediation. From the perspective of different soil points (P1–P9) and electric field regions (cathode, middle, and anode), the Cd content, chemical speciation redistribution characteristics of contaminated red soil have been analyzed after long-term non-closed cycles (150 d for 10.5 cycles), periodic reversal DC EK, and EKPR treatment.

**Figure 3:**
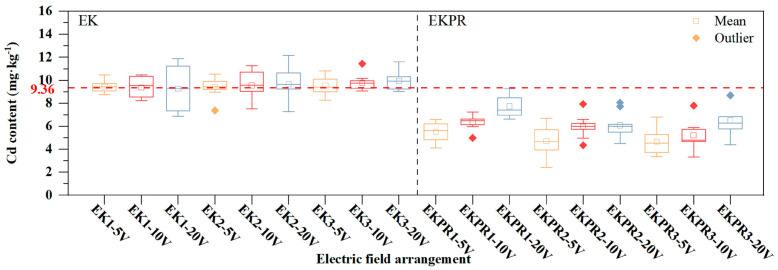
Box plot of Cd content under different treatments.

Figure 3 shows that in EK1, EK2, and EK3, the average soil Cd residue values, ranging from 9.26 to 9.96 mg·kg^−1^, were only slightly different from the initial value of 9.36 mg·kg^−1^. This finding indicates that there is no significant removal effect of EK under direct connection conditions, and the Cd content of the surface soil in the multidimensional (2D, 3D) electric field increases. Table S4 reveals that the Cd content in the surface layer (0–6 cm) is greater than that in the bottom layer (6–12 cm) in 3D electric fields, mainly because the perpendicular direction of the electric field promotes the migration of Cd^2+^ from the bottom soil to the surface soil through electromigration. This is in line with the results of previous studies [16,22]. However, in EKPR, the soil Cd content was lower than the initial value, and the removal rate was 0.4% to 50.32%. When comparing EK and EKPR, the main reason for the decrease in soil Cd content was due to the uptake of Cd by *S. plumbizincicola*. At the same electric field dimension, the Cd removal rate decreased with an increase in the electric field dimension (EKPR1 and 2, 5–20 V). While at the same voltage, the removal rate increased with an increase in the electric field dimension (5 and 10 V, EKPR1-3. Figure 3). There are two reasons for this phenomenon. Firstly, the magnitude of the voltage affects the growth of *S. plumbizincicola* and, thus, its Cd uptake. Secondly, the multidimensional electric field generates denser electric field lines, which enable the accumulation of more heavy metals toward the root-accessible area and promote the uptake of Cd by *S. plumbizincicola*. The distribution range and quartiles of Cd content in different treatments varied with the electric field, indicating that the spatial distribution anisotropy of soil Cd may be related to the electric field layout.

Table 1 shows the distribution of Cd at points P1–P9 under various treatments. In contrast to the uniform distribution of Cd in the initial soil, the distribution of Cd in each treatment is uneven and shows regularity. In EK1, the Cd content at points P1, P4, and P7 is higher than that at points P3, P6, and P9, and the disparity becomes more pronounced as the voltage rises. In EK2 and EK3, the Cd content at point P5 is higher than that of the others. Similarly, all the aforementioned points are arranged in the cathode region. Soil Cd migrated from the anode to the middle and cathode (with the cathode region > the anode region) and accumulated in the middle and cathode regions [10]. The relative migration rate (RMr) was adopted to assess the migration degree of Cd, and there exist certain differences among the different treatments (from 5 V to 10 V, in the cathode regions: EK1 −0.92–23.98%, EK2 5.11–18.02%, EK3 7.52–11.52%; in the middle regions: EK1 −3.37–9.64%, EK2 1.70–12.30%, EK3 −0.42–6.68%). The RMr in the cathode region is greater than that in the middle region. Under the same electric field dimension, the RMr in both regions increases with the increase in voltage. Under the same voltage, the RMr in both regions decreases with the increase in the electric field dimension. The more uneven distribution of soil Cd and higher overall Cd content under the multidimensional electric field compared to the 1D electric field may be caused by the dense electric field lines (in the vertical direction) of the multidimensional electric field, which migrate the bottom Cd to the surface layer. The surface soil was collected for Cd content determination [10]. In EKPR, the relationship between RMr and voltage, as well as the electric field dimension, was not obvious, which might be attributed to the plant growth and the uptake of Cd. Nevertheless, compared with the EK treatment, the Cd content at each point in EKPR decreased significantly. A reduction in Cd ranging from 0.94% to 64% was observed under EKPR treatment compared to the original soil Cd, with the lowest soil Cd content being 3.37 mg⋅kg^−1^ (EKPR2–5 V P3). The accumulation of Cd by *S. plumbizincicola* is conspicuous, and the removal law of Cd is 5 V > 10 V > 20 V.

**Figure 4:**
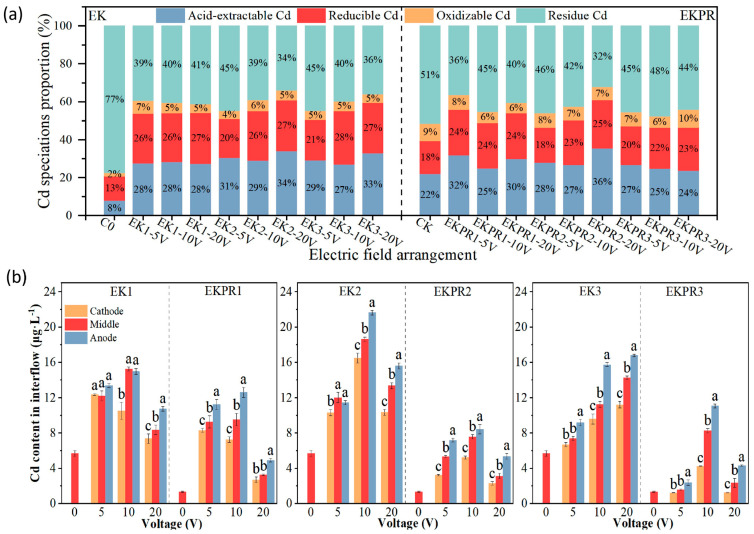
(**a**) The proportion of Cd speciations under different treatments (the average proportion of four speciations of Cd content at each point); (**b**) Cd content of pore water in the cathode–anode regions under different treatments. (Bars represent means ± S.E. of three independent replicates. Those not sharing the same letters are significantly different at *p* < 0.05, as determined by Duncan’s multiple range tests.)

The removal efficiency of Cd has a significant connection to the Cd speciation. Acid-extractable Cd is easily absorbed, while residue Cd is the most stable and not easily absorbed [34]. Figure 4a shows that the original soil (C0) Cd mainly exists in the residue state, and the content accounts for as high as 77%, which may be the reason why Cd is difficult to remove in the actual contaminated soil. For the single plant action (CK), residue Cd accounted for 51%, a decrease of approximately 26% compared to C0. The proportions of the remaining three speciations of Cd increased to a certain extent, with acid-extractable Cd increasing by about 14%. The plant may do so through the secretion of low molecular weight organic acids to activate Cd and improve the bioavailability of Cd [31,35]. In EK, the percentages of both acid-extractable Cd and reduced Cd increased significantly, and the percentage of residue Cd decreased significantly. The percentage of acid-extractable Cd increased by 19–26% compared to C0, and by 5–12% compared to CK. The effect of a single electric field can increase the content of acid-extractable Cd in the soil, and its performance is better than that of a single plant. Additionally, in EK2 and EK3, the acid-extractable Cd was higher than that in EK1, and the residue Cd decreased as the voltage increased. These results indicate that the electric field effect can be used to change the soil physicochemical properties, thus contributing to the release of heavy metals in an acid-extractable state to the soil environment [15,21]. On the other hand, the multidimensional electric field will cause the electromigration of Cd from the bottom layer to the surface layer, leading to an increase in the content of acid-extractable Cd [10]. Acid-extractable Cd in EKPR was reduced compared with that in EK, but it was significantly higher than in CK. The change in the percentage of each speciation was not obvious, which may be related to the absorption of acid-extractable Cd from the soil by *S. plumbizincicola* in EKPR.

The distribution of Cd fractions in samples P1–P9 under different treatments is presented in Figure S1 (Supplementary Materials). For most sampling sites, the Cd fraction content followed the order: residual Cd > reducible Cd > acid-extractable Cd > oxidizable Cd. Notably, the content of acid-extractable and residual Cd is the key factor influencing Cd accumulation in plants. The following results focus on the analysis and discussion of these two Cd speciations. In EK1 (5–20 V), the acid-extractable Cd content (P1–P9: 5 V ranging from 2.24 to 2.86 mg⋅kg^−1^, 10 V ranging from 2.42 to 2.95 mg⋅kg^−1^, 20 V ranging from 2.20 to 3.08 mg⋅kg^−1^), the higher the voltage, the greater the difference in acid-extractable Cd content at each point. The content of acid-extractable Cd at points P1, P4, and P7 was lower than that at points P3, P6, and P9, while the content of residue Cd was the opposite. This may be due to the decrease in pH in the anode region of the 1D electric field (P3, P6, P9) and the increase in acid-extractable Cd content. Compared with EK1, this pattern is not obvious in EKPR1, but the contents of acid-extractable Cd and residue Cd decrease significantly, which is attributed to the absorption by plants. In EK2 (5–20 V), the content of acid-extractable Cd changed significantly along with the voltage, and the content of acid-extractable Cd (4.10 mg⋅kg^−1^) in EK2–20 V P5 reached the maximum. Cd migrated from the anode region (P1, P3, P4, P6, P7, P9) to the cathode region (P5) through electromigration and electrodialysis. In EK3 (5–20 V), the content of residue Cd decreased, and the content of acid-extractable Cd increased significantly. Compared with EK and EKPR, the content of acid-extractable Cd was absorbed by plants, and the content of residue Cd, reduced Cd, and acid-extractable Cd decreased to a certain extent. Compared with EK2, EK3, and EK1, regarding the content and distribution of Cd speciations, the larger the electric field dimension, the greater the difference.

Soil Cd desorption directly influences the migration and morphological transformation of Cd in soil, thus affecting the efficiency of plant Cd extraction. The Cd content in soil pore water can directly reflect the desorption of Cd. Figure 4b shows the Cd content in soil pore water under different treatments. The Cd content in soil pore water increased in the electric field. In EK, the soil pore water Cd content rose by 17.78–227.82%, with a maximum of 21.63 μg·L^−1^ (EK2–10 V anode). Compared to EK, the variation rule of the soil pore water Cd content with the voltage under the EKPR treatment was not evident. By comparing C0 with CK and EK with EKPR, plants would significantly reduce the soil Cd content through uptake accumulation. The Cd content of pore water in the anode region was significantly higher than that in the cathode region. However, the difference in Cd content of pore water in the cathode and anode regions under the three electric fields at 5 V and 20 V was less than at 10 V, which may be caused by both direct and indirect effects of the electric field. Some studies have found that application of a DC electric field can directly promote the desorption of metals from soil particles to soil pore water, and the desorbed heavy metals will undergo electrodialysis and electromigration [36,37], which makes the spatial distribution of soil heavy metals different, and Cd is similar to it in this experiment. Furthermore, soil pH affects Cd desorption, while anode precipitation hydrogen acidification facilitates soil Cd desorption, and anode soil pore water Cd content increases. However, the Cd desorbed from the anode migrated to the cathode in the electric field, resulting in the difference in soil pore water Cd content between the two regions. Meanwhile, the results of this study showed that the difference in Cd desorption was more significant in EKPR compared with that in EK, the purely electrically mediated system, which may also be related to the regulation of plant inter–root secretions [35].

### 3.3. Plant Growth and Cd Accumulation

**Figure 5:**
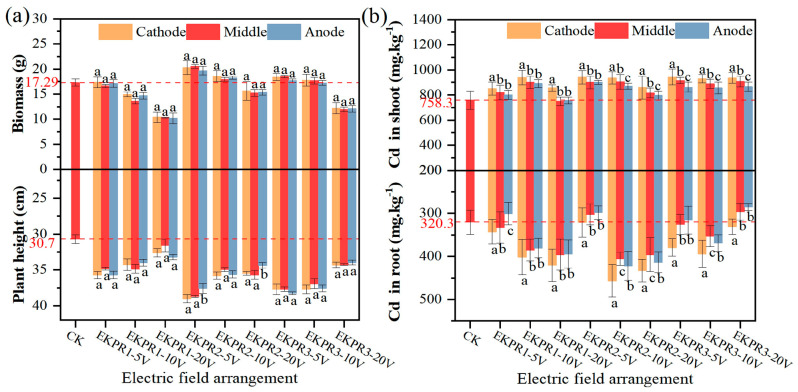
(**a**) The growth situation of *Sedum plumbizincicola* in the cathode–anode regions; (**b**) the Cd uptake of *S. plumbizincicola* in the cathode–anode regions. (Bars represent means ± S.E. of three independent replicates. Those not sharing the same letters are significantly different at *p* < 0.05, as determined by Duncan’s multiple range tests.)

The effect of an electric field on the growth of *Sedum plumbizincicola* is shown in Figure 5a. The biomass of *S. plumbizincicola* was higher than that of CK at 5 V, with an increase of 0.4–17.12%, which promoted the growth of *S. plumbizincicola*, while it was lower than that of CK at 20 V, with a decrease of 9.6–39.8%, which inhibited the growth of *S. plumbizincicola*. The biomass of *S. plumbizincicola* followed the order of 5 V > 10 V > 20 V under the same electric field configuration, and EKPR2 > EKPR3 > EKPR1 under the same voltage. The height of *S. plumbizincicola* was promoted in EKPR, which was increased by 6.2–29.3% compared to that in CK. The height of *S. plumbizincicola* followed the trend: 5 V > 10 V > 20 V. The differences in the biomass and the height of *S. plumbizincicola* were not obvious in the different soil regions in EKPR with polarity reversal DC electric fields. Previous studies have found that low voltage (such as 0.2 V·cm^−1^ for *Lolium perenne* L. and 1 V⋅cm^−1^ for *Brassica juncea*) will promote their growth, while high voltage will inhibit their growth [29,38]. In this experiment, the growth of *S. plumbizincicola* was more sensitive to the electric field. It was also promoted under the action of a low-voltage electric field (5 V and 10 V), while growth was inhibited under a high-voltage electric field (20 V). The low-voltage electric field promotes plant growth for three reasons: first, the electric field in the physicochemical characteristics of the soil and the local concentration of contaminants and nutrients [18]; second, electroosmosis and electromigration increase the opportunity for plant roots to absorb soluble ions [39]; third, it improves nutrient uptake and enhances plant metabolism by stimulating ion fluxes and ion channels in the root membrane [40]. The increase in below-ground biomass has increased the potential for plant root uptake and accumulation of heavy metals [41]. Figure 5b shows that the Cd accumulation in shoot and root of *S. plumbizincicola* increased in EKPR by 4.80–24.29% and 1.77–42.52%, respectively, compared with that of CK (decreased in some regions under some treatments, e.g., EKPR1–20 V), and the law of Cd accumulation in shoot was 5 V > 20 V > 10 V in EKPR2 and EKPR3. The Cd transport factor (TF) and bioconcentration factor (BCF) of *S. plumbizincicola* were as shown in Table S5. The maximum TF was 3.06 (EKPR3–20 V, middle), and the TF increased significantly in EKPR2–5 V and EKPR3–20 V. The Cd BCF of the *S. plumbizincicola* was increased by −5.51–108.48% with a maximum of 214.80 (EKPR3–5 V, cathode). In conclusion, the biomass of the *S. plumbizincicola*, did not differ zonally in the electric field but only had an effect on the overall biomass. However, the accumulation behavior of *S. plumbizincicola* to Cd was significantly different in different regions, following that of cathode > anode.

The overall growth, Cd accumulation, and soil Cd removal rate of *S. plumbizincicola* under each treatment are shown in Table 2. The design of the electric field had a great influence on the growth and Cd accumulation efficiency of *S. plumbizincicola*. Plant growth was promoted in the appropriate voltage range (5 V and 10 V), and plant growth was inhibited by too high a voltage (20 V). For the electric field arrangement, the multidimensional electric field has denser electric field lines compared with the 1D electric field, which can more easily promote the migration of Cd, thus solving the problem of the limited plant root range. Polarity reversal in electrode orientation can prevent extreme changes in soil pH. In this experiment, the growth of *S. plumbizincicola*, Cd accumulation, and soil Cd removal were unsatisfactory at 20 V. The biomass decreased by 40%, and the soil Cd removal was only 17% in EKPR1–20 V. The growth of *S. plumbizincicola*, Cd accumulation, and soil Cd removal were the highest under EKPR2–5 V. The biomass increased by 16.6%, Cd accumulation increased by 29%, and soil Cd removal was as high as 50%.

### 3.4. Cd Removal Mechanism in the EKPR System

**Figure 6:**
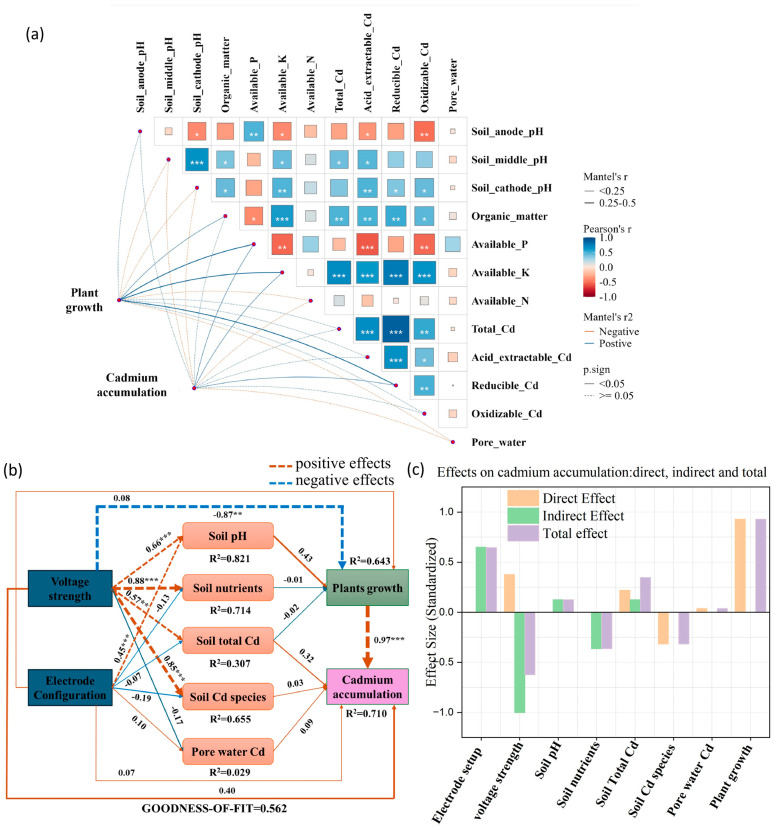
(**a**) Correlation analysis heatmap of soil properties, Cd speciation characteristics, and plant growth traits with Cd accumulation in the EKPR system. (The area of each square is proportional to the absolute value of the Pearson correlation coefficient (*r*), with larger squares representing stronger correlations (either positive or negative)) (**b**) Structural equation model. (Line thickness and corresponding numerical values indicate the magnitude of the effect, with thicker lines and larger values representing stronger effects); ** (*p* < 0.01); *** (*p* < 0.001). (**c**) Effect decomposition diagram. (*, indicates a significant correlation at the 0.05 level (*p* < 0.05), **, indicates a significant correlation at the 0.01 level (*p* < 0.01), ***, indicates a significant correlation at the 0.001 level (*p* < 0.001)).

Figure 6 shows the Cd removal mechanism in the EKPR system. Figure 6a quantifies the linear correlation strength and significance among soil physicochemical properties, Cd speciation, plant growth traits, and Cd accumulation under EKPR conditions. The heatmap revealed that available soil nutrients exhibited a significant and strong positive correlation with acid-extractable Cd under the EKPAR remediation system (correlation coefficient r > 0.7, *p* < 0.001). This phenomenon could be attributed to the enhanced competitive adsorption between nutrient ions and Cd ions on the surface of soil colloids driven by the electrokinetic effect, which further promoted the conversion of Cd into the more bioavailable acid-extractable fraction [42]. pH exhibited a significant negative correlation with plant growth and acid-extractable Cd (r < −0.6, *p* < 0.01). This phenomenon pertains to the fundamental mechanism of electrokinetic remediation. As the electrodynamic force drives H^+^ migration toward the cathode, it significantly reduces the pH of the anodic zone soil, wherein anodic electrolysis results in the generation of H^+^ ions. As electrodynamic forces drive H^+^ migration toward the cathode, the pH of the anodic soil zone decreases. In acidic environments, H^+^ ions can displace Cd ions adsorbed on soil colloids via ion exchange, increasing the proportion of acid-extractable Cd. Additionally, acidic conditions facilitate the uptake and translocation of mineral nutrients and Cd ions by plant roots, thereby promoting plant growth [43]. Plant growth traits (biomass, plant height) under electrodynamic remediation showed a highly significant positive correlation with Cd accumulation (r ≈ 0.95, *p* < 0.001). This phenomenon may be attributed to electrodynamic restoration promoting plant growth, resulting in more developed root systems. The organic acids secreted by these plants further activate Cd in the rhizosphere, thereby increasing the available form of Cd in the soil. Additionally, the application of electric force enhances the activity of root ion transporters, significantly improving the plant’s uptake of acid-extractable Cd from the soil and its transport efficiency to the aboveground parts [44,45]. Moreover, no discernible biomass dilution effect was observed under electrodynamic regulation in this study, with Cd uptake rates exceeding biomass growth rates, further reinforcing their positive correlation, confirming that plant growth status under electrokinetic regulation is a pivotal factor in controlling Cd accumulation. It is noteworthy that the correlation between soil total Cd and plant Cd accumulation was relatively weak (r < 0.3, *p* > 0.05), indicating that it is the bioavailability (morphological characteristics) of Cd under electrokinetic remediation rather than its total content that is being regulated.

Figure 6b presents the structural equation model (SEM. The unit of analysis was an independent potted/field remediation plot (*N* = 30, data sourced from clean_data.csv). Data independence was ensured through non-duplicate samples, spatial isolation, and variance inflation factor (VIF) testing for multicollinearity. The model comprised 9 reflective latent variables and 19 observed variables with no missing data, following the hypothesized path “electrodynamics → soil properties → plant growth/Cd accumulation”. Parameter estimation adopted the default path weighting scheme with a convergence threshold of 1 × 10^−8^, and path diagrams were visualized using the innerplot() function. Standard fit indices were reported as follows: goodness-of-fit (GOF = 0.562), average variance extracted (AVE = 0.859), coefficient of determination (R^2^ = 0.396), standardized root-mean-square residual (SRMR = 0.565), and predictive relevance (Q^2^ = 0.475), confirming the rationality of the core paths.), which further clarifies the direct and indirect pathways through which electrode configuration and voltage intensity influence soil properties, plant growth, and Cd accumulation within the EKPR remediation system. Exogenous variables (voltage intensity, electrode configuration) indirectly influence plant growth and Cd accumulation by regulating mediating variables such as soil pH, nutrients, and Cd speciation. The SEM results are consistent with the hypothesized pathway: soil nutrients and soil pH both exert significant regulatory effects on plant growth, with distinct differences in the direction and intensity of their action. Soil nutrients show a significant positive driving effect (path coefficient = 0.66, *p* < 0.01), while soil pH exerts a significant negative regulatory effect (path coefficient = −0.58, *p* < 0.01). Collectively, these two factors form the core pathway for the regulation of plant growth by soil physicochemical properties in the hypothesized structure. In contrast to the significant effects of soil physicochemical properties, Cd forms, total soil Cd, and pore water Cd all failed to exert significant direct effects on Cd accumulation (absolute path coefficients < 0.2, *p* > 0.05). And the path coefficient between total soil Cd and Cd accumulation approached zero. This further corroborates the conclusion in Figure (a) that total soil Cd has a weak correlation with plant Cd accumulation. Moreover, plant growth exerts an extremely strong direct positive driving effect on Cd accumulation (path coefficient 0.97, *p* < 0.001). It acts as the core mediating pathway through which soil physicochemical properties and Cd-related factors regulate Cd accumulation. Notably, the coefficient of determination (R^2^) for each variable shows that the model explains 82.1% of the variation in soil pH. It also explains the variation in plant growth and Cd accumulation, thus validating the model’s goodness of fit (GOF = 0.562). This proves the model is reliable in revealing the core driving pathways of Cd accumulation in the EKPR system. Figure 6c presents an effect decomposition diagram. It quantifies the pathways in Figure 6b into direct, indirect effects, and total effects, and intuitively shows the combined influence characteristics of each factor on Cd accumulation. Voltage intensity exerts a dominant negative indirect effect on Cd accumulation (effect magnitude ≈ −0.35), with no significant direct effect. The negative indirect effect of electrode configuration on Cd accumulation is comparable to that of voltage intensity. It similarly acts through the soil properties–plant growth pathway. Plant growth exerts extremely strong positive direct effects (≈0.97) and total effects (≈0.98) on Cd accumulation, further confirming its role as the core driver. Factors such as soil pH and total Cd content show total effects approaching zero. This indicates their positive and negative effects cancel each other out, resulting in negligible overall influence.

In summary, Figure 6 progressively elucidates the core mechanism of Cd removal within the EKPR remediation system through a tiered approach of correlation linkage–pathway mechanism–effect decomposition: Electrodynamic forces indirectly influence plant growth and Cd uptake capacity by regulating soil pH, nutrients, and Cd speciation. Plant growth serves as the pivotal link driving Cd accumulation (and consequently Cd removal). This provides a clear theoretical basis and technical target for optimizing EKPR remediation parameters and enhancing the remediation efficiency of cadmium-contaminated soils.

## 4. Conclusions

The current study shows that the variation of soil pH was proportional to the applied voltage (0–20 V). No extreme acidification or alkalization occurred (5.01–7.03 and 4.35–7.15 for EKPR and EK system of 20 V, respectively), alleviated by periodic polarity reversal and the plant *S. plumbizincicola.* After CK, EK, and EKP treatments, the residual fraction of soil Cd was transformed into other fractions. The degree of influence on Cd speciation conversion followed the order of EKPR ≈ EK > CK. This trend was particularly pronounced for the acid-extractable and reducible fractions. EK could promote the dissociation of Cd into soil pore water (a 17.78–227.82% increase in different regions with a range of 5–20 V). The Cd content in the soil pore water of the EK system was substantially higher than that of the EKPR system. This phenomenon is attributed to the efficient phytoextraction of *S. plumbizincicola*. The migration of Cd from the anode to the cathode and from the bottom to the surface (2D and 3D) is caused by the electric field. Under the synergistic effect of direct interface action and indirect influence (such as soil pH, EC changes) of the electric field, soil Cd can continuously dissociate, migrate, and accumulate circularly in the middle and cathode regions. In comparison with biomass, the Cd accumulation in the shoot and TF, the height of *S. plumbizincicola*, Cd accumulation in the root, and BCF increased significantly with an increase in the voltage range of 0–10 V (significant inhibition at 20 V). The suitable electric field arrangement and voltage for simultaneously promoting the growth and Cd accumulation of *S. plumbizincicola* were 2D and 5 V, respectively. And the biomass, Cd accumulation, and soil Cd removal rate were 18.21 g·pot^−1^, 0.98 g·kg^−1^, and 50%, improved by 16.59%, 29.31%, and 28.76% compared with CK, respectively. Through correlation heatmaps, structural equation modeling, and effect decomposition diagrams, electrodynamics indirectly influences plant growth and Cd accumulation by regulating soil pH, nutrients, and Cd speciation. Plant growth serves as the key driver enabling Cd accumulation for remediation, providing theoretical and technical targets for optimizing remediation parameters and enhancing efficiency. In summary, electrodynamics promotes the growth of *S. plumbizincicola*, Cd accumulation, and Cd removal via the EKPR system by regulating soil physicochemical properties, Cd speciation, distribution, dissociation, and bioavailability.

## Figures and Tables

**Table 1:** Distribution of soil Cd content in P1–P9 points under different treatments.

Different Treatments	Voltage	P1	P2	P3	P4	P5	P6	P7	P8	P9
EK1	5 V	9.7 ± 0.8	9.1 ± 0.6	9.6 ± 0.6	9.7 ± 0.4	8.8 ± 0.1	9.1 ± 0.8	9.2 ± 1.0	9.4 ± 0.4	10.5 ± 0.7
	10 V	10.5 ± 0.8	8.7 ± 0.3	8.4 ± 0.3	10.5 ± 0.6	9.5 ± 0.6	8.3 ± 0.9	10.3 ± 0.8	9.6 ± 0.5	8.5 ± 0.2
	20 V	11.9 ± 1.3	8.4 ± 0.3	7.3 ± 1.2	11.2 ± 0.5	9.3 ± 0.4	7.3 ± 1.0	11.9 ± 0.5	9.3 ± 0.9	6.9 ± 0.2
EK2	5 V	10.6 ± 0.3	9.9 ± 0.9	9.4 ± 1.5	9.6 ± 0.3	10.2 ± 0.7	9.4 ± 0.8	9.0 ± 0.7	9.2 ± 0.1	7.4 ± 0.4
	10 V	10.7 ± 0.6	10.8 ± 0.4	9.6 ± 0.6	9.0 ± 0.9	11.3 ± 0.3	7.9 ± 0.1	9.8 ± 0.9	9.6 ± 0.4	7.5 ± 0.4
	20 V	9.2 ± 0.5	10.6 ± 0.3	9.7 ± 0.4	9.9 ± 0.1	12.1 ± 0.3	7.3 ± 0.5	9.2 ± 1.2	11.5 ± 0.3	7.4 ± 0.2
EK3	5 V	10.1 ± 0.6	9.2 ± 1	8.3 ± 0.6	9 ± 0.4	10.8 ± 0.4	8.5 ± 1.2	9.8 ± 1.1	9.4 ± 0.9	10.7 ± 1.5
	10 V	10.2 ± 0.5	9.8 ± 1.1	9.1 ± 0.6	9.3 ± 0.2	11.4 ± 0.2	9.3 ± 0.8	10.0 ± 0.9	9.7 ± 1.4	9.3 ± 1.5
	20 V	9.3 ± 0.8	10.3 ± 1.6	9.0 ± 0.1	9.9 ± 1.4	11.6 ± 0.6	10.0 ± 0.5	9.2 ± 0.7	11.1 ± 0.3	9.2 ± 0.6
EKPR1	5 V	6.5 ± 0.9	6.2 ± 1.0	4.1 ± 0.6	5.6 ± 1.2	6.1 ± 1.2	5.0 ± 0.8	6.6 ± 1.0	4.8 ± 1.2	4.6 ± 0.8
	10 V	6.4 ± 1.5	6.9 ± 0.7	6.5 ± 2.0	6.5 ± 1.6	6.0 ± 1.5	7.3 ± 0.6	6.6 ± 1.5	5.0 ± 0.7	6.1 ± 1.4
	20 V	8.6 ± 1.2	7.4 ± 0.8	7.0 ± 1.0	9.3 ± 0.9	8.1 ± 1.5	7.1 ± 1.3	8.5 ± 1.2	6.6 ± 0.8	6.9 ± 1.4
EKPR2	5 V	6.7 ± 0.5	4.6 ± 0.9	2.4 ± 0.5	3.8 ± 1.4	3.9 ± 1.1	4.8 ± 0.6	6.3 ± 0.1	4.1 ± 1.1	5.7 ± 1.5
	10 V	6.0 ± 0.9	7.9 ± 1.6	4.3 ± 1.5	5.7 ± 0.9	6.6 ± 0.7	6.2 ± 1.6	6.2 ± 0.7	5.7 ± 0.7	5.0 ± 1.7
	20 V	4.5 ± 1.3	7.7 ± 1.1	6.0 ± 1.5	6.0 ± 1.1	6.2 ± 1.2	5.5 ± 1.6	5.3 ± 1.3	8.1 ± 1.0	5.5 ± 0.9
EKPR3	5 V	5.2 ± 0.9	4.5 ± 0.8	3.4 ± 0.4	3.5 ± 0.8	3.7 ± 0.6	3.8 ± 0.3	5.8 ± 0.6	6.8 ± 1.1	5.3 ± 0.8
	10 V	4.7 ± 0.9	5.7 ± 1.4	3.3 ± 0.5	4.8 ± 0.7	7.8 ± 0.6	4.2 ± 0.4	5.7 ± 0.8	5.9 ± 0.3	4.7 ± 0.6
	20 V	8.7 ± 0.9	5.8 ± 1.1	4.4 ± 0.5	6.4 ± 0.7	5.9 ± 0.7	8.7 ± 0.2	5.6 ± 0.6	6.8 ± 1.5	6.3 ± 0.3

**Table 2:** The growth, Cd accumulation, and Cd removal efficiency of the overall *Sedum plumbizincicola* under different treatments.

Remediation Systems	Voltage	Biomass (g·pot^−1^)	Cd Accumulation Within the Plant (g·kg^−1^)	Soil Cd Remove Rate (%)
EKPR1	5 V	17.24 ± 0.29	0.82 ± 0.02	41%
	10 V	20.16 ± 0.36	0.91 ± 0.02	32%
	20 V	14.41 ± 0.60	0.79 ± 0.05	17%
EKPR2	5 V	10.32 ± 0.08	0.98 ± 0.1	50%
	10 V	18.26 ± 0.25	0.82 ± 0.03	36%
	20 V	15.41 ± 0.17	0.80 ± 0.03	35%
EKPR3	5 V	18.21 ± 0.37	0.91 ± 0.03	50%
	10 V	17.57 ± 0.24	0.89 ± 0.03	44%
	20 V	12.01 ± 0.1	0.90 ± 0.03	31%

## Data Availability

The original contributions presented in this study are included in the article/Supplementary Material. Further inquiries can be directed to the corresponding author.

## Supplementary Materials

The following supporting information can be downloaded at: https://www.mdpi.com/article/10.3390/plants15030481/s1, Table S1. Basic chemical characteristics of the soil. Table S2. EKPR experiment layout plan. Table S3. Soil pH in the surface and bottom layers of the cathode and anode regions under three electric field with 5 V. Table S4. Soil Cd in the Surface and Bottom layers of the cathode and anode regions under three electric field with 5 V. Table S5. Translocation factor and bioconcentration factor of *Sedum plumbizincicola*. Figure S1. The content of Cd speciations in soil of each point under different treatments.

## References

[1] Song P., Xu D., Yue J., Ma Y., Dong S., Feng J. (2022). Recent Advances in Soil Remediation Technology for Heavy Metal Contaminated Sites: A Critical Review. Sci. Total Environ..

[2] Wu H., Tong J., Jiang X., Wang J., Zhang H., Luo Y., Pang J., Shi J. (2024). More Effective than Direct Contact: Nano Hydroxyapatite Pre-Treatment Regulates the Growth and Cd Uptake of Rice (*Oryza sativa* L.) Seedlings. J. Hazard. Mater..

[3] Liu F., Liu X., Ding C., Wu L. (2015). The Dynamic Simulation of Rice Growth Parameters under Cadmium Stress with the Assimilation of Multi-Period Spectral Indices and Crop Model. Field Crops Res..

[4] Wang Y., Tan R., Zhou L., Lian J., Wu X., He R., Yang F., He X., Zhu W. (2021). Heavy Metal Fixation of Lead-Contaminated Soil Using *Morchella mycelium*. Environ. Pollut..

[5] Hu W., Niu Y., Zhu H., Dong K., Wang D., Liu F. (2021). Remediation of Zinc-Contaminated Soils by Using the Two-Step Washing with Citric Acid and Water-Soluble Chitosan. Chemosphere.

[6] Liu L., Li W., Song W., Guo M. (2018). Remediation Techniques for Heavy Metal-Contaminated Soils: Principles and Applicability. Sci. Total Environ..

[7] Zhong X., Chen Z., Li Y., Ding K., Liu W., Liu Y., Yuan Y., Zhang M., Baker A.J.M., Yang W. (2020). Factors Influencing Heavy Metal Availability and Risk Assessment of Soils at Typical Metal Mines in Eastern China. J. Hazard. Mater..

[8] Ali H., Khan E., Sajad M.A. (2013). Phytoremediation of Heavy Metals—Concepts and Applications. Chemosphere.

[9] Wang J., Aghajani Delavar M. (2024). Modelling Phytoremediation: Concepts, Methods, Challenges and Perspectives. Soil Environ. Health.

[10] Fan G., Zhou D., Zhang Z., Ai Y., Zhang W., Shi G., Tong F., Liu L., Chen W., Li J. (2021). Effect of Two-Dimensional Electric Field on the Growth and Cadmium Uptake of *Sedum plumbizincicola*. Sep. Purif. Technol..

[11] Zhang J., Na M., Wang Y., Ge W., Zhou J., Zhou S. (2024). Cadmium Levels and Soil pH Drive Structure and Function Differentiation of Endophytic Bacterial Communities in *Sedum plumbizincicola*: A Field Study. Sci. Total Environ..

[12] Sarwar N., Imran M., Shaheen M.R., Ishaque W., Kamran M.A., Matloob A., Rehim A., Hussain S. (2017). Phytoremediation Strategies for Soils Contaminated with Heavy Metals: Modifications and Future Perspectives. Chemosphere.

[13] Ma H., Duan Z., Guo J., Zhu X., Shi X., Zhou W., Jiang M., Xiong J., Li T. (2022). Lead Dissociation and Redistribution Properties of Actual Contaminated Farmland Soil after Long-Term EKAPR Treatment. Environ. Geochem. Health.

[14] Cai Z., Sun Y., Deng Y., Zheng X., Sun S., Romantschuk M., Sinkkonen A. (2021). In situ electrokinetic (EK) remediation of the total and plant available cadmium (Cd) in paddy agricultural soil using low voltage gradients at pilot and full scales. Sci. Total Environ..

[15] Chen Y., Dong M., Lyu P., Wang A., Wang H., Li J. (2023). Analysis of metal(loid) pollution and possibilities of electrokinetic phytoremediation of abandoned coking plant soil. Sci. Total Environ..

[16] Cang L., Wang Q., Zhou D., Xu H. (2011). Effects of Electrokinetic-Assisted Phytoremediation of a Multiple-Metal Contaminated Soil on Soil Metal Bioavailability and Uptake by Indian Mustard. Sep. Purif. Technol..

[17] Sánchez V., López-Bellido F.J., Rodrigo M.A., Fernández F.J., Rodríguez L. (2020). A Mesocosm Study of Electrokinetic-Assisted Phytoremediation of Atrazine-Polluted Soils. Sep. Purif. Technol..

[18] Putra R.S., Ohkawa Y., Tanaka S. (2013). Application of EAPR System on the Removal of Lead from Sandy Soil and Uptake by Kentucky Bluegrass (*Poa pratensis* L.). Sep. Purif. Technol..

[19] Cameselle C., Gouveia S. (2019). Phytoremediation of Mixed Contaminated Soil Enhanced with Electric Current. J. Hazard. Mater..

[20] Li J., Zhang J., Larson S.L., Ballard J.H., Guo K., Arslan Z., Ma Y., Waggoner C.A., White J.R., Han F.X. (2019). Electrokinetic-Enhanced Phytoremediation of Uranium-Contaminated Soil Using Sunflower and Indian Mustard. Int. J. Phytoremediat..

[21] Mao X., Han F.X., Shao X., Su Y. (2015). Coupled Electro-Kinetic Remediation and Phytoremediation of Metal(Loid) Contaminated Soils. J. Bioremediat. Biodegrad..

[22] Luan Y.J., Xu J.Z., Li Y.W., Hu Z.W., Wang H.Y., Wang Y.H., Xu X.H. (2024). Polarized Switching Electric Field-Assisted Phytoremediation of Cadmium-Contaminated Paddy Soil. Trans. Chin. Soc. Agric. Mach..

[23] Luo J., Xing X., Qi S., Wu J., Gu X.W.S. (2019). Comparing the Risk of Metal Leaching in Phytoremediation Using *Noccaea caerulescens* with or without Electric Field. Chemosphere.

[24] Han D., Wu X., Li R., Tang X., Xiao S., Scholz M. (2021). Critical Review of Electro-Kinetic Remediation of Contaminated Soils and Sediments: Mechanisms, Performances and Technologies. Water. Air. Soil Pollut..

[25] Vocciante M., Bagatin R., Ferro S. (2017). Enhancements in ElectroKinetic Remediation Technology: Focus on Water Management and Wastewater Recovery. Chem. Eng. J..

[26] Li X., Yang Z., He X., Liu Y. (2020). Optimization Analysis and Mechanism Exploration on the Removal of Cadmium from Contaminated Soil by Electrokinetic Remediation. Sep. Purif. Technol..

[27] Serghei A., Tress M., Sangoro J.R., Kremer F. (2009). Electrode Polarization and Charge Transport at Solid Interfaces. Phys. Rev. B.

[28] Kartal Ş., Aydın Z., Tokalıoğlu Ş. (2006). Fractionation of Metals in Street Sediment Samples by Using the BCR Sequential Extraction Procedure and Multivariate Statistical Elucidation of the Data. J. Hazard. Mater..

[29] Huang K., Sun X., Sun J., Guo Y., Hu X., Hu C., Tan Q. (2023). The Role of Phosphorus Speciation of Biochar in Reducing Available Cd and Phytoavailability in Mining Area Soil: Effect and Mechanism. Sci. Total Environ..

[30] Shen X., Li C., Li M., Zhou K., Li Y. (2021). Effect of Electric Potentials on the Removal of Cu and Zn in Soil by Electrokinetic Remediation. Sep. Sci. Technol..

[31] Xu Y., Xu X., Hou H., Zhang J., Zhang D., Qian G. (2016). Moisture Content-Affected Electrokinetic Remediation of Cr(VI)-Contaminated Clay by a Hydrocalumite Barrier. Environ. Sci. Pollut. Res..

[32] Sun X., Li Z., Wu L., Christie P., Luo Y., Fornara D.A. (2019). Root-Induced Soil Acidification and Cadmium Mobilization in the Rhizosphere of *Sedum plumbizincicola*: Evidence from a High-Resolution Imaging Study. Plant Soil.

[33] Luo J., Wu J., Huo S., Qi S., Gu X.S. (2018). A Real Scale Phytoremediation of Multi-Metal Contaminated e-Waste Recycling Site with Eucalyptus Globulus Assisted by Electrical Fields. Chemosphere.

[34] Wang Y. (2022). Effects and Mechanisms of Plant Root Exudates on Soil Remediation. Acta Ecol. Sin..

[35] Wang P., Peng H., Liu J., Zhu Z., Bi X., Yu Q., Zhang J. (2020). Effects of Exogenous Dissolved Organic Matter on the Adsorption–Desorption Behaviors and Bioavailabilities of Cd and Hg in a Plant–Soil System. Sci. Total Environ..

[36] Raeisi S., Motaghian H., Hosseinpur A.R. (2020). Effect of the Soil Biochar Aging on the Sorption and Desorption of Pb^2+^ under Competition of Zn^2+^ in a Sandy Calcareous Soil. Environ. Earth Sci..

[37] Tahmasbian I., Safari Sinegani A.A. (2013). Monitoring the Effects of Chelating Agents and Electrical Fields on Active Forms of Pb and Zn in Contaminated Soil. Environ. Monit. Assess..

[38] Acosta-Santoyo G., Cameselle C., Bustos E. (2017). Electrokinetic—Enhanced Ryegrass Cultures in Soils Polluted with Organic and Inorganic Compounds. Environ. Res..

[39] Chang J.-H., Dong C.-D., Shen S.-Y. (2019). The Lead Contaminated Land Treated by the Circulation-Enhanced Electrokinetics and Phytoremediation in Field Scale. J. Hazard. Mater..

[40] Mao X., Han F.X., Shao X., Guo K., McComb J., Arslan Z., Zhang Z. (2016). Electro-Kinetic Remediation Coupled with Phytoremediation to Remove Lead, Arsenic and Cesium from Contaminated Paddy Soil. Ecotoxicol. Environ. Saf..

[41] Cang L., Zhou D.-M., Wang Q.-Y., Fan G.-P. (2012). Impact of Electrokinetic-Assisted Phytoremediation of Heavy Metal Contaminated Soil on Its Physicochemical Properties, Enzymatic and Microbial Activities. Electrochim. Acta.

[42] Zhang M.L., Long L.-L., Zhu Q.-R., Chen C., Xu M., Wu J., Yang G. (2024). Mechanism and ecological environmental risk assessment of peroxymonosulfate for the treatment of heavy metals in soil. Sci. Total Environ..

[43] Fang L., Rong-Bing F., Zhen X. (2015). Optimization of electrode configuration in soil electrokinetic remediation. Huan Jing Ke Xue Huanjing Kexue.

[44] Bu A., Yao G., Zhou C., Mao Z., Liu B., Ma J., Fang X., Liu D., Ye Z. (2024). Effect of AC electric field on enhancing phytoremediation of Cd-contaminated soils in different pH soils. Sci. Rep..

[45] Fan L., Yuan Q., Lu Q., Zheng C., Su R., Liu N., Wu J. (2024). Remediation of cadmium contaminated soil using electrokinetic-phytoremediation system with rotary switching electrodes. Environ. Geochem. Health.

