# Supplementary material for: Effects of Electric Field Dimensions on Electrokinetically Enhanced Cadmium Dissociation and Phytoremediation in Plateau Red Soil
Source: Plants (Basel). 2026 Feb 4;15(3):481. doi: 10.3390/plants15030481 (PMC12899018; doi:10.3390/plants15030481)
Supplement: Supplementary file 1 [file plants-15-00481-s001.zip › plants-4062917-supplementary.pdf]

Supporting information

Table S1 Basic chemical characteristics of the soil.

pH	Organic matter ($g\ kg^{-1}$)	Alkeline–N ($mg\ kg^{-1}$)	Olsen–P ($mg\ kg^{-1}$)	Available–K ($mg\ kg^{-1}$)	Pb ($mg\ kg^{-1}$)	Cd ($mg\ kg^{-1}$)
5.45	19.1	65.3	23.6	317	366	9.36

Table S2 EKPR experiment layout plan.

**Voltage (V)**	**Layout scheme**							**Amount**
	No plants (EK)				With plants (EKPR)			
	CK	EK1	EK2	EK3	EKPR1	EKPR2	EKPR3	
**0**	CK	/	/	/	/	/	/	3
**5**	/	EK1–5	EK2–5	EK3–5	EKPR1–5	EKPR2–5	EKPR3–5	18
**10**	/	EK1-10	EK2-10	EK3-10	EKPR1-10	EKPR2-10	EKPR3-10	18
**20**	/	EK1–20	EK2–20	EK3–20	EKPR1–20	EKPR2–20	EKPR3–20	18
**Amount**	3	9	9	9	9	9	9	57

Table S3 Soil pH in the surface and bottom layers of the cathode and anode regions under three electric field with 5 V.

Electrical fields	level	EK			EKPR		
		Cathode	Middle	Anode	Cathode	Middle	Anode
1D	Surface	5.90 ± 0.02	5.91 ± 0.13	5.63 ± 0.07	6.07 ± 0.04	5.93 ± 0.06	5.59 ± 0.13
	Bottom	5.76 ± 0.13	5.77 ± 0.17	5.41 ± 0.19	6.02 ± 0.15	5.83 ± 0.14	5.54 ± 0.13
2D	Surface	6.20 ± 0.03	5.81 ± 0.04	5.56 ± 0.34	5.9 ± 0.13	5.79 ± 0.05	5.52 ± 0.21
	Bottom	4.87 ± 0.02	4.82 ± 0.04	4.62 ± 0.35	4.73 ± 0.05	4.60 ± 0.04	4.43 ± 0.20
3D	Surface	5.51 ± 0.03	5.64 ± 0.04	5.75 ± 0.31	5.78 ± 0.01	5.78 ± 0.04	5.43 ± 0.19
	Bottom	4.47 ± 0.06	5.53 ± 0.04	5.85 ± 0.28	4.46 ± 0.05	5.44 ± 0.04	5.93 ± 0.17

Table S4 Soil Cd in the Surface and Bottom layers of the cathode and anode regions under three electric field with 5 V.

Electrical fields	level	EK			EKPR		
		Cathode	Middle	Anode	Cathode	Middle	Anode
1D	Surface	6.98 ± 0.86	7.35 ± 0.75	6.64 ± 1.66	6.22 ± 0.52	5.72 ± 0.78	4.57 ± 0.42
	Bottom	7.77 ± 0.68	8.43 ± 0.18	8.01 ± 1.29	6.60 ± 0.58	6.26 ± 0.71	5.16 ± 0.70
2D	Surface	2.42 ± 0.05	8.31 ± 0.87	8.98 ± 0.82	3.94 ± 0.32	4.38 ± 0.37	4.94 ± 1.62
	Bottom	2.63 ± 0.32	9.12 ± 0.59	9.26 ± 0.81	3.02 ± 0.15	5.52 ± 0.68	4.93 ± 0.59
3D	Surface	5.18 ± 0.12	8.93 ± 1.36	9.43 ± 1.11	3.72 ± 0.31	5.65 ± 1.61	4.49 ± 1.04
	Bottom	4.2 ± 0.96	7.40 ± 0.08	8.95 ± 0.78	2.24 ± 0.10	4.43 ± 0.51	4.98 ± 0.60

Table S5 Translocation factor and bioconcentration factor of *Sedum plumbizincicola*.

**Electric field layout**	**TF (Translocation factor)**			**BCF (Bioconcentration factor)**		
	**Cathode**	**Middle**	**Anode**	**Cathode**	**Middle**	**Anode**
**CK**	/	2.37	/	/	103.03	/
**EKPR1–5 V**	2.48	2.47	2.67	136.55	179.45	175.13
**EKPR1-10 V**	2.34	2.34	2.34	144.83	135.81	134.25
**EKPR1–20 V**	2.04	1.89	1.92	97.35	107.08	108.01
**EKPR2–5 V**	2.93	2.98	3.02	214.80	182.22	182.24
**EKPR2-10 V**	2.05	2.23	2.06	137.19	162.36	155.85
**EKPR2–20 V**	1.99	2.06	1.92	108.98	149.21	145.54
**EKPR3–5 V**	2.48	2.81	2.73	166.91	204.05	192.19
**EKPR3-10 V**	2.36	2.52	2.32	160.48	194.19	187.18
**EKPR3–20 V**	2.82	3.06	3.04	148.60	135.77	129.74

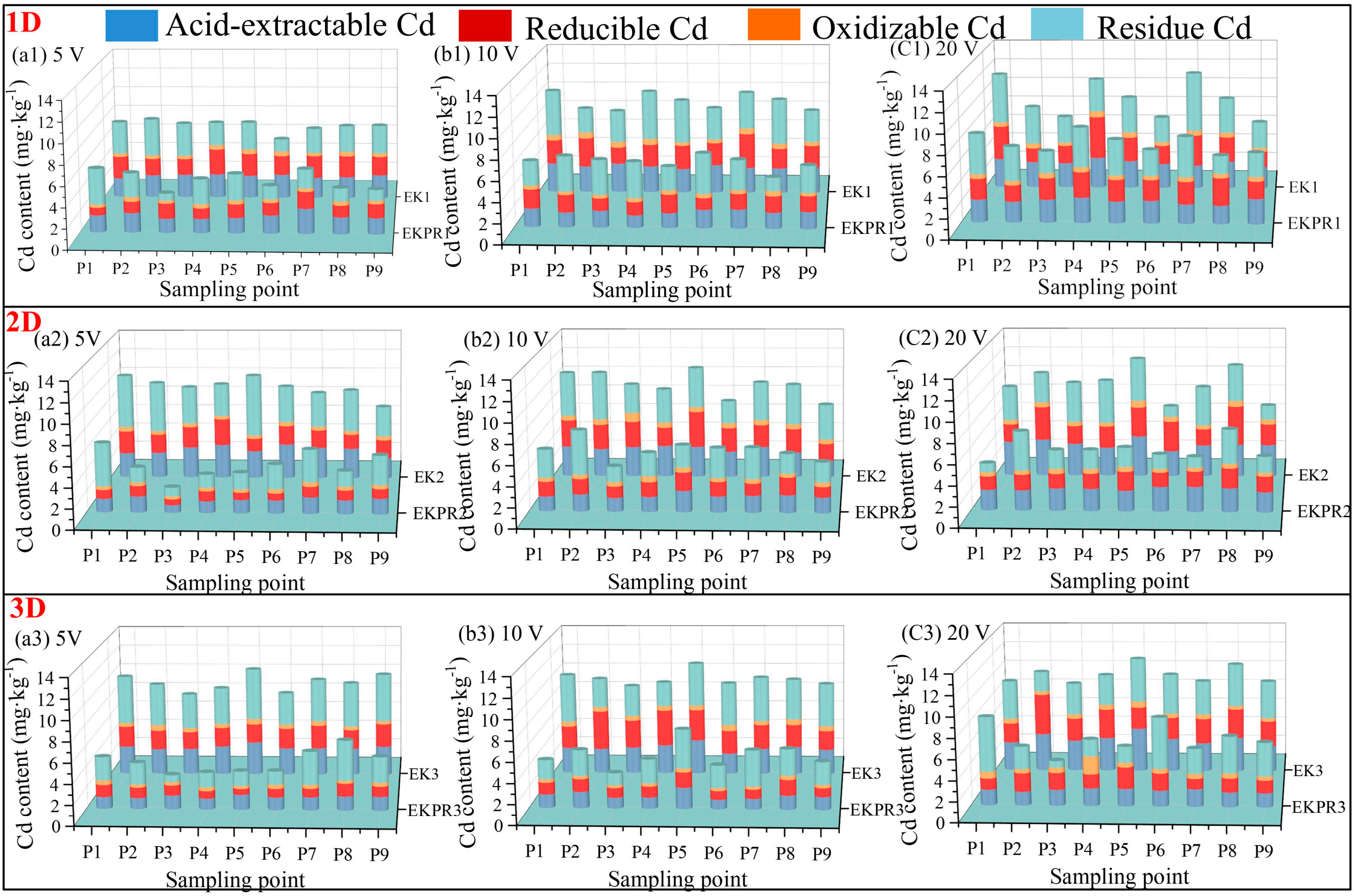

Figure S1.The content of Cd speciations in soil of each point under different treatments.
